# Humoral immune responses to hyaluronan oligosaccharides in patients undergoing prosthetic valve surgery

**DOI:** 10.3389/fimmu.2026.1762676

**Published:** 2026-02-18

**Authors:** Daniel Bello-Gil, Sara Olivera-Ardid, Arnau Blasco-Lucas, Fabrizio Sbraga, Manuel Galiñanes, Thierry Le Tourneau, Jean-Christian Roussel, Tomaso Bottio, Marta Vadori, Emanuele Cozzi, Cesare Galli, Cristina Costa, Vered Padler-Karavani, Jean-Paul Soulillou, Rafael Mañez

**Affiliations:** 1 Infectious Diseases and Transplantation Division, Institut d’Investigació Biomèdica de Bellvitge (IDIBELL), L’Hospitalet de Llobregat, Barcelona, Spain; 2 Cardiac Surgery Department, Bellvitge University Hospital, L’Hospitalet de Llobregat, Barcelona, Spain; 3 Department of Cardiac Surgery and Reparative Therapy of the Heart, Vall d’Hebron Research Institute (VHIR), University Hospital Vall d’Hebron, Universitat Autònoma de Barcelona, Barcelona, Spain; 4 Institut du Thorax, Institut National de la Santé et de la Recherche Médicale Unité Mixte de Recherche 1087 (UMR1087), University Hospital, Nantes, France; 5 Cardiovascular Regenerative Medicine Group, Department of Cardiac, Thoracic and Vascular Surgery, University of Padova, Padova, Italy; 6 Transplantation Immunology Unit, Padua University Hospital, Padova, Italy; 7 Avantea, Cremona, Italy; 8 Department of Cell Research and Immunology, Shmunis School of Biomedicine and Cancer Research, George S. Wise Faculty of Life Sciences, Tel Aviv University, Tel Aviv, Israel; 9 Institut de Transplantation-Urologie-Néphrologie, Institut National de la Santé et de la Recherche Médicale Unité Mixte de Recherche 1064, Centre Hospitalier Universitaire de Nantes, Nantes, France; 10 Intensive Care Department, Bellvitge University Hospital, L’Hospitalet de Llobregat, Barcelona, Spain

**Keywords:** antiglycan antibodies, anti-hyaluronan antibodies, biological heart valves, cardiac surgery, hyaluronan, hyaluronan oligosaccharides, printed glycan array

## Abstract

**Background:**

Bioprosthetic heart valves (BHVs) are widely used in cardiac surgery but are limited by structural valve deterioration (SVD). The Translink study showed that immune responses to xenogeneic glycans contribute to SVD. Hyaluronan (HA), a glycosaminoglycan with size-dependent biological functions, has been implicated in inflammation, xenoreactivity, and valve calcification. This study investigated humoral immune responses to HA oligosaccharides in a subgroup of the Translink cohort.

**Patients and methods:**

Serum samples from 258 BHV recipients and 78 cardiac surgery controls were analyzed by ELISA, and a representative subset was further profiled using a printed glycan array (PGA). BHV recipients were classified with clinically significant SVD (A), as *de novo* implantation (B1), or long-term without SVD (B2). Controls included mechanical valve replacement or coronary artery bypass patients, either *de novo* (B1C) or long-term post-surgery (B2C). ELISA quantified IgG and IgM antibodies against HA fragments of increasing length (HA2, HA24, HA84), while PGA mapped fine specificity across HA2–HA40.

**Results:**

All subjects exhibited measurable baseline anti-HA IgG and IgM antibodies. ELISA revealed a fragment-length dependence, with the shortest fragment, HA2, showing the highest antibody binding, and progressively lower reactivity with longer oligomers. Following *de novo* cardiac surgery, both BHV recipients and controls showed a transient increase in anti-HA antibodies, peaking at one month and declining within 6–12 months. In contrast, PGA consistently identified HA34 as the dominant immunogenic fragment across all cohorts and time points.

**Conclusions:**

Anti-HA antibodies are constitutively present in humans, undergo transient amplification after cardiac surgery, regardless of prosthesis type, and display marked fragment-length specificity. Short HA2 fragments predominate in ELISA responses, whereas intermediate-length oligomers, such as HA34, emerge as conserved, immunodominant targets in PGA. These findings extend the Translink paradigm from xenogeneic to matrix-derived glycan immunity and identify anti-HA antibodies as potential biomarkers of postoperative inflammation and tissue remodeling, which could be relevant to SVD.

## 1 Introduction

Compared with mechanical prostheses, bioprosthetic heart valves (BHVs) are increasingly used to manage valvular heart disease because of their favorable hemodynamic performance and lower thromboembolic risk, as they do not require lifelong anticoagulation (1). These advantages are particularly relevant for elderly patients and for those with contraindications to anticoagulation. However, the major limitation of BHVs is their restricted durability, as structural valve deterioration (SVD) typically emerges within 10–15 years after implantation (2), often necessitating reoperation or transcatheter reintervention. The mechanisms underlying SVD are multifactorial and incompletely understood (3, 4), but increasing evidence suggests an immune response against xenogeneic antigens (5).

Among the most intensively studied xenoantigens are the carbohydrate epitopes galactose-α1,3-galactose (αGal) and N-glycolylneuraminic acid (Neu5Gc) (6, 7). Both are widely expressed in animal-derived tissues used for BHV manufacturing but are not expressed in humans (8, 9). Preexisting natural antibodies against αGal and Neu5Gc have long been recognized as barriers to xenotransplantation, and the multicenter Translink study recently confirmed their role in BHV degeneration (5). In this study, antibody responses against αGal and Neu5Gc increased following BHV implantation, and their presence in explanted valves was associated with calcification and degeneration.

While αGal and Neu5Gc represent prototypical glycan xenoantigens, other extracellular matrix (ECM)-derived glycans may also elicit immune responses in the context of BHV implantation. Hyaluronan (HA), a ubiquitous glycosaminoglycan in the extracellular matrix (ECM) of all mammalian tissues, has been implicated in native cardiac valve calcification and xenoreactivity (10, 11). HA is a linear polysaccharide composed of repeating disaccharide units of glucuronic acid (GlcA) and N-acetylglucosamine (GlcNAc), and its biological effects are strongly oligosaccharide size dependent (12). High–molecular–weight HA contributes to tissue homeostasis, hydration, and structural integrity, whereas fragmented HA acts as a damage-associated molecular pattern (DAMP), promoting inflammation by activating macrophages and dendritic cells and by engaging signaling receptors such as TLR2, TLR4, and CD44 (13, 14). These pathways induce the production of proinflammatory mediators, including TNFα, IL-1β, IL-6, and matrix metalloproteinases, which can amplify tissue injury and remodeling. Experimental evidence also suggests that extracellular HA, through CD44-mediated signaling, can promote calcification of native aortic valves (10). As calcification is central to SVD, HA-directed immune responses may represent an underappreciated pathway linking ECM remodeling to BHV deterioration.

The immunogenicity of HA has been demonstrated in experimental systems. Animal studies have shown that rabbits and rats develop anti-HA antibodies following exposure to streptococci or liposome-bound HA, with antibody titers enhanced by enzymatic digestion of HA, implicating HA oligosaccharides as the principal antigenic targets (15, 16). More recently, anti-HA antibodies have been detected in nonhuman primates and rodents, and their titers have been shown to increase after xenotransplantation (11). Human data are scarce, with only a single report describing elevated anti-HA2 and anti-HA24 antibodies in preeclamptic pregnancies compared with healthy controls (17).

Based on these observations, the present study was designed to test two related hypotheses. First, we hypothesized that exposure to xenogeneic bioprosthetic material would preferentially enhance humoral immune responses to HA compared with non-xenogeneic cardiac surgery controls. Second, we hypothesized that HA immunogenicity in humans is primarily determined by fragment length rather than by HA per se, with specific oligomer sizes selectively targeted by circulating antibodies.

To distinguish xenogeneic effects from surgery-related immune activation, patients undergoing mechanical heart valve replacement or coronary artery bypass grafting were included as control groups. Although these procedures do not involve xenogeneic tissue, they are associated with substantial tissue injury, ischemia–reperfusion stress, and ECM remodeling, all of which can generate HA fragments that can stimulate anti-HA antibody responses. Inclusion of these control cohorts, therefore, allows assessment of whether HA-directed humoral immunity is specific to bioprosthetic valve implantation or reflects a more general consequence of cardiac surgery–induced matrix disruption.

Building on the Translink study framework, which established antiglycan immunity as a contributing factor to BHV outcomes, the present investigation focused on antibodies against HA oligosaccharides in a well-defined subgroup of the same patient population. Combining ELISA and printed glycan array (PGA) technologies, we aimed to characterize the presence, dynamics, and fragment-length specificity of anti-HA antibodies in BHV recipients compared with those in other cardiac surgery controls.

## 2 Material and methods

### 2.1 Translink cohort and study groups

The prospective, multicenter Translink study (ClinicalTrials.gov NCT02023970) enrolled 1,426 patients receiving BHV and 242 controls undergoing other cardiac surgeries. Its overall design and primary results have been previously reported (5). The study protocol was reviewed and approved by the European Commission Seventh Framework Programme (FP7) Ethics Committee and the ethics committees of the four hospitals participating in sample collection: the Nantes University Hospital in France; the Bellvitge University Hospital of the Catalan Health Institute in Barcelona, Spain; the University Hospital Vall D’Hebron of the Catalan Health Institute in Barcelona, Spain; and the Azienda Ospedaliera di Padova in Padova, Italy. All clinical investigations were conducted in accordance with the 2013 Declaration of Helsinki. All study participants provided written informed consent to participate in the trial.

The subgroup analysis of the present study included 258 BHV recipients and 78 controls, sampled for HA-directed antibodies. BHV recipients were stratified as follows: Group A (n=29), clinically significant SVD at sampling; Group B1 (n=133), at *de novo* BHV implantation; and Group B2 (n=96), ≥4 years post-implantation without clinically significant SVD. The controls were B1C (n=43), patients who underwent mechanical valve replacement (MHV) or coronary artery bypass grafting (CABG) alongside B1, and B2C (n=35), long-term surgical controls who underwent MHV or CABG ≥4 years ago, enrolled alongside B2. A comparable number of samples from each of the four participating centers was included in each clinical group, with selection determined by the chronological order in which eligible samples became available and without prespecification based on clinical status, antibody levels, or outcomes.

### 2.2 Blood sampling and handling

The sampling schedules varied according to patients’ clinical status. Group A: at SVD diagnosis. Groups B1 and B1C: pre-surgery (baseline) and at 1, 6, 12, and 24 months post-surgery. Groups B2 and B2C: at recruitment (>48 months post-BHV) and again 12–24 months later (>60–72 months). Sera were prepared via standard protocols and stored at –80 °C until analysis.

### 2.3 ELISA for anti-HA antibodies

Anti-HA antibodies were quantified via ELISA as previously described (11). The number of samples analyzed by ELISA was primarily determined by the availability and batch consistency of HA–PAA conjugates (HA2, HA24, and HA84), as well as by serum volume and sample integrity. Because the amount of HA84–PAA was more limited, antibodies against this fragment were assessed only in Groups B1 and B1C. In addition, serum samples from Groups B2 and B2C at 12 months were insufficient in volume for parallel ELISA and PGA analyses and were therefore excluded from ELISA at this time point.

HA fragments were synthesized from hyaluronic acid by reductive amination with 2-(4-aminophenyl)ethylamine (APEA) and sodium cyanoborohydride, yielding HA-APEA glycamines. For ELISA, 96-well Maxisorp plates were coated overnight at 4 °C with 0.5 µg/well HA-polyacrylamide (PAA) conjugates representing three HA fragment lengths (HA2, the disaccharide unit; HA24, containing 12 disaccharide units; and HA84, containing 42 disaccharide units) in carbonate buffer (pH 9.6). PAA without HA served as a negative control. The plates were blocked with 1% BSA in PBS-Tween, and diluted serum samples (1:100) were added in duplicate. After incubation and washing, the bound antibodies were detected via horseradish peroxidase–conjugated goat anti-human antibodies (1:4000). Color development was achieved with an o-phenylenediamine (OPD) substrate, which was stopped with 3 N HCl, and the absorbance was measured at 492 nm.

### 2.4 Printed glycan array for fine specificity of anti-HA antibodies

PGA spanning HA2–HA40 (1–20 repeating disaccharide units) was used to measure the fine specificity of human anti-HA antibodies, as previously described (11). The number of samples analyzed by the PGA was determined by the availability of the array and the serum volume. Samples from all participating centers were included, with selection based on the chronological order of sample availability and without prespecified clinical or serological selection criteria. Hyaluronic acid was enzymatically digested with bovine testicular hyaluronidase to generate small oligomers, which were conjugated to the bifunctional fluorescent linker AEAB and purified by reverse-phase ion-pairing HPLC using water/acetonitrile with dibutylamine and acetic acid. The purified HA fragments were dried, reconstituted in water, and lyophilized before microarray printing. Using a noncontact Aushon2470 Arrayer, HA glycans were printed at 100 µM in sodium phosphate buffer (pH 8.5) to produce 16 identical subarrays per slide, each with six replicates per glycan. The slides were blocked with sodium ethanolamine in sodium borate and stored at –20 °C until use. Serum samples (1:15 dilution in PBS with 1% BSA and 1% Tween-20) were incubated on the slides at 37 °C for 1.5 h, followed by sequential washes and incubation with biotin-conjugated goat anti-IgG/IgM/IgA secondary antibodies (1:200). After washing, Cy5-labeled streptavidin (1:500) was added. The slides were incubated in the dark, washed, and air-dried. The fluorescence signals were acquired via a ScanArray GX Plus scanner (excitation 633 nm), and the data were analyzed via ScanArrayR Express software. The results are expressed as relative fluorescence units (RFUs) reported as the median ± median absolute deviation (MAD). The results from replicated spots were averaged and log-transformed (base 2) to improve interpretability and visualization. Heatmaps were used to summarize and visualize PGA antibody reactivity profiles across HA fragment lengths and time points.

### 2.5 Statistics

Continuous variables are summarized as median and range unless otherwise stated. All statistical tests were two-sided, and p-values <0.05 were considered statistically significant. Statistical analyses were performed using GraphPad Prism v9.5.1 (GraphPad Software, San Diego, CA, USA).

To compare anti-hyaluronan antibody levels at the initial sampling time point across the five cohorts (A, B1, B1C, B2, and B2C), a Kruskal–Wallis test was applied. *Post hoc* pairwise comparisons were performed using Dunn’s multiple-comparison correction. The Mann-Whitney test was used to compare the initial anti-HA84 IgG and IgM antibody levels between Groups B1 and B1C.

Longitudinal changes in anti-HA antibody levels in Groups B1 and B1C were analyzed separately using linear mixed-effects models, with time as a fixed effect and subject as a random effect to account for repeated measures and missing values. *Post hoc* pairwise comparisons among all time points were performed using Tukey’s multiple-comparisons test, with adjustment for multiple comparisons. ELISA values were log10-transformed to stabilize variance after adding a small constant (1) to accommodate zero values.

## 3 Results

### 3.1 Baseline anti-HA antibodies across study groups by ELISA

**Figure 1:**
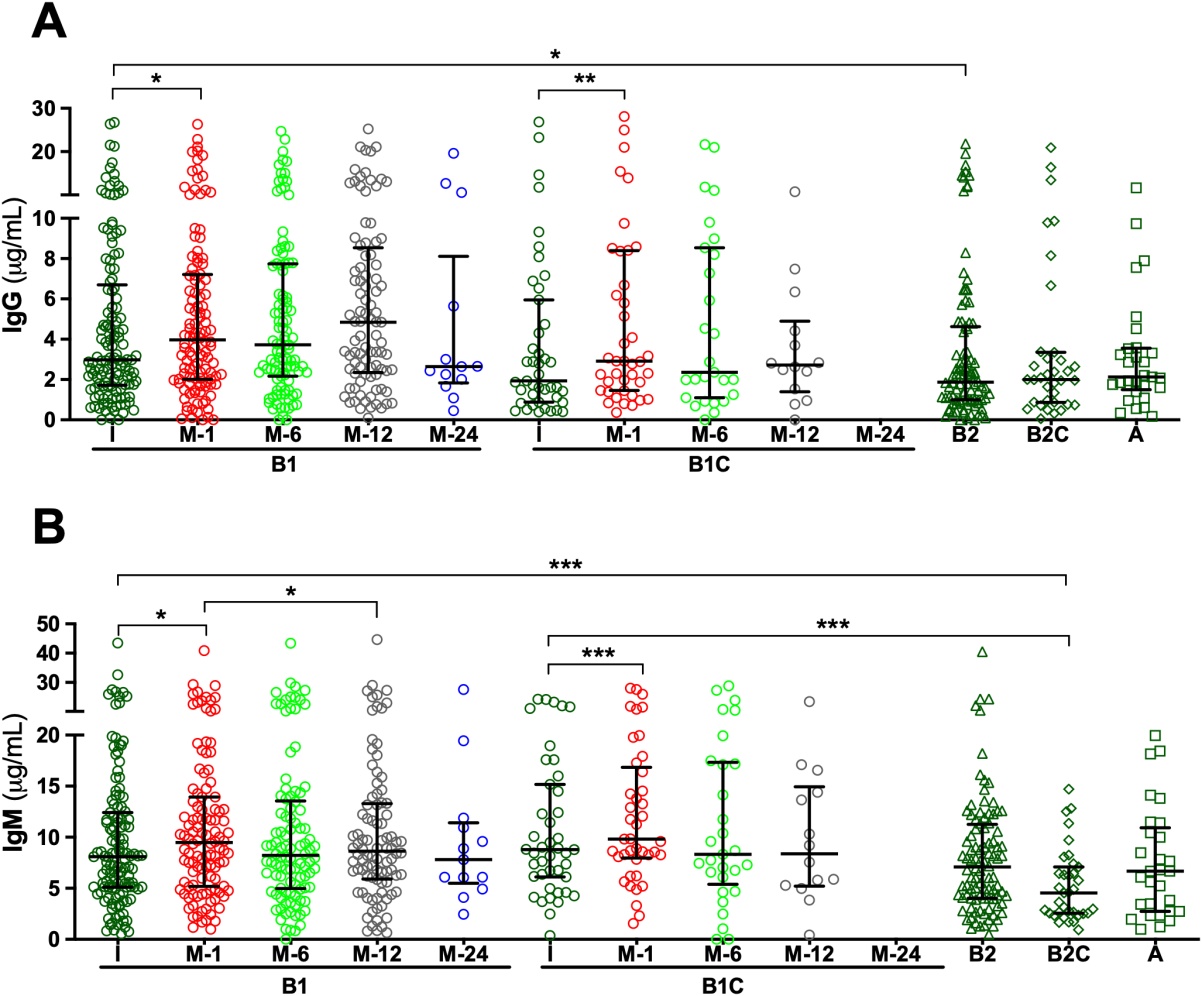
Serum antibodies against the disaccharide unit of hyaluronan (HA2) measured via ELISA. **(A)** IgG antibodies. **(B)** IgM antibodies. Longitudinal analyses are shown for cohorts B1 (*de novo* BHV implantation) and B1C (*de novo* MHV or CABG), with serum samples collected before surgery (I) and at 1, 6, 12, and 24 months post-operatively (M-1, M-6, M-12, and M-24). For cohorts B2 (≥4 years post-BHV without SVD), B2C (≥4 years post-MHV implantation or CABG), and A (SVD), only the initial sample obtained at study inclusion was analyzed. The results are expressed in μg/mL as median values with interquartile ranges (25th–75th percentiles). (*p < 0.05; **p < 0.01; ***p < 0.001).

**Figure 2:**
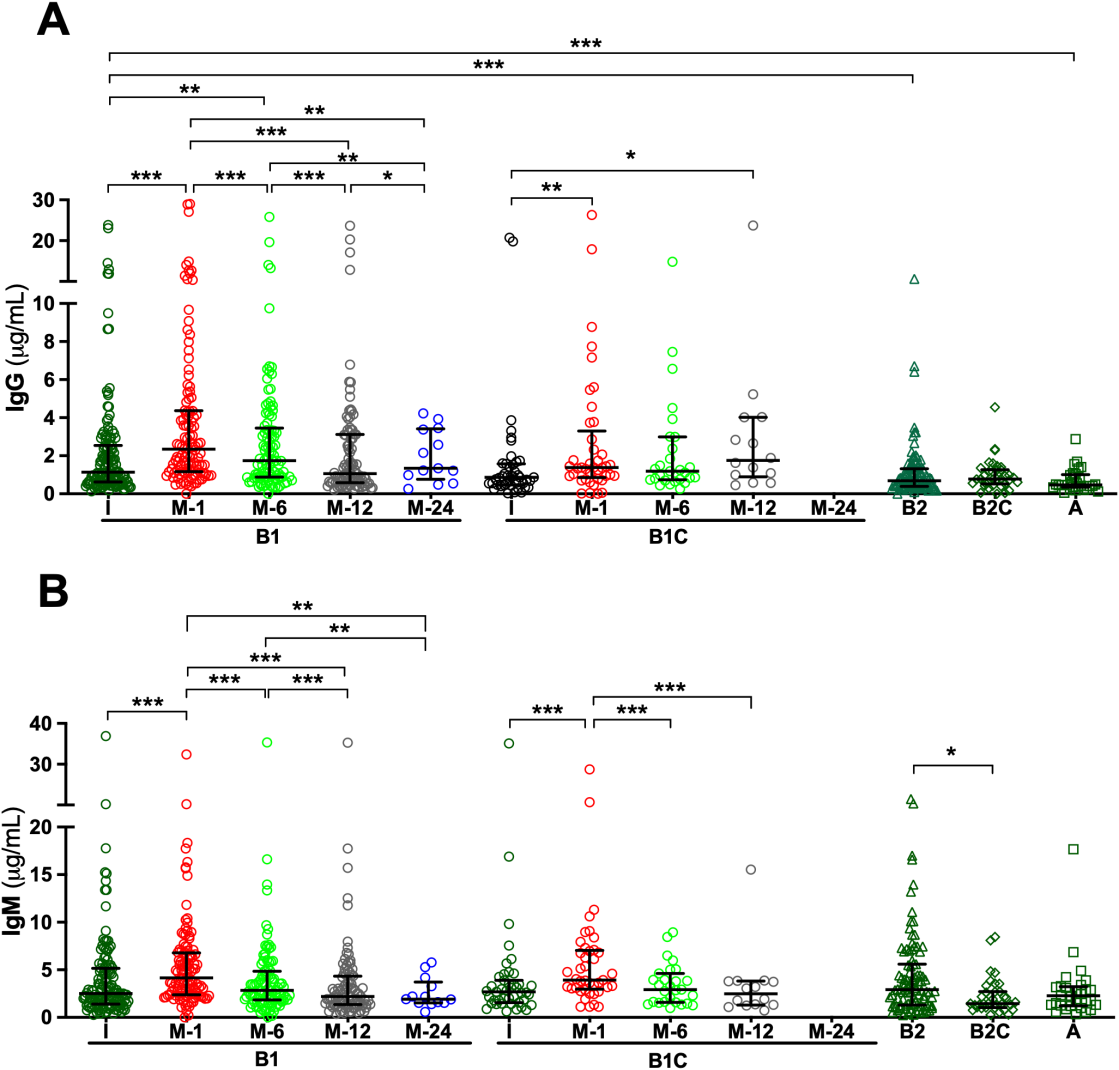
Serum antibodies against the hyaluronan fragment HA24 (12 disaccharide units) measured by ELISA. **(A)** IgG antibodies. **(B)** IgM antibodies. Longitudinal analyses are shown for cohorts B1 (*de novo* BHV implantation) and B1C (*de novo* MHV or CABG), with serum samples collected before surgery (I) and at 1, 6, 12, and 24 months post-operatively (M-1, M-6, M-12, and M-24). For cohorts B2 (≥4 years post-BHV without SVD), B2C (≥4 years post-MHV implantation or CABG), and A (SVD), only the initial sample obtained at study inclusion was analyzed. The results are expressed in μg/mL as median values with interquartile ranges (25th–75th percentiles). (*p < 0.05; **p < 0.01; ***p < 0.001).

**Figure 3:**
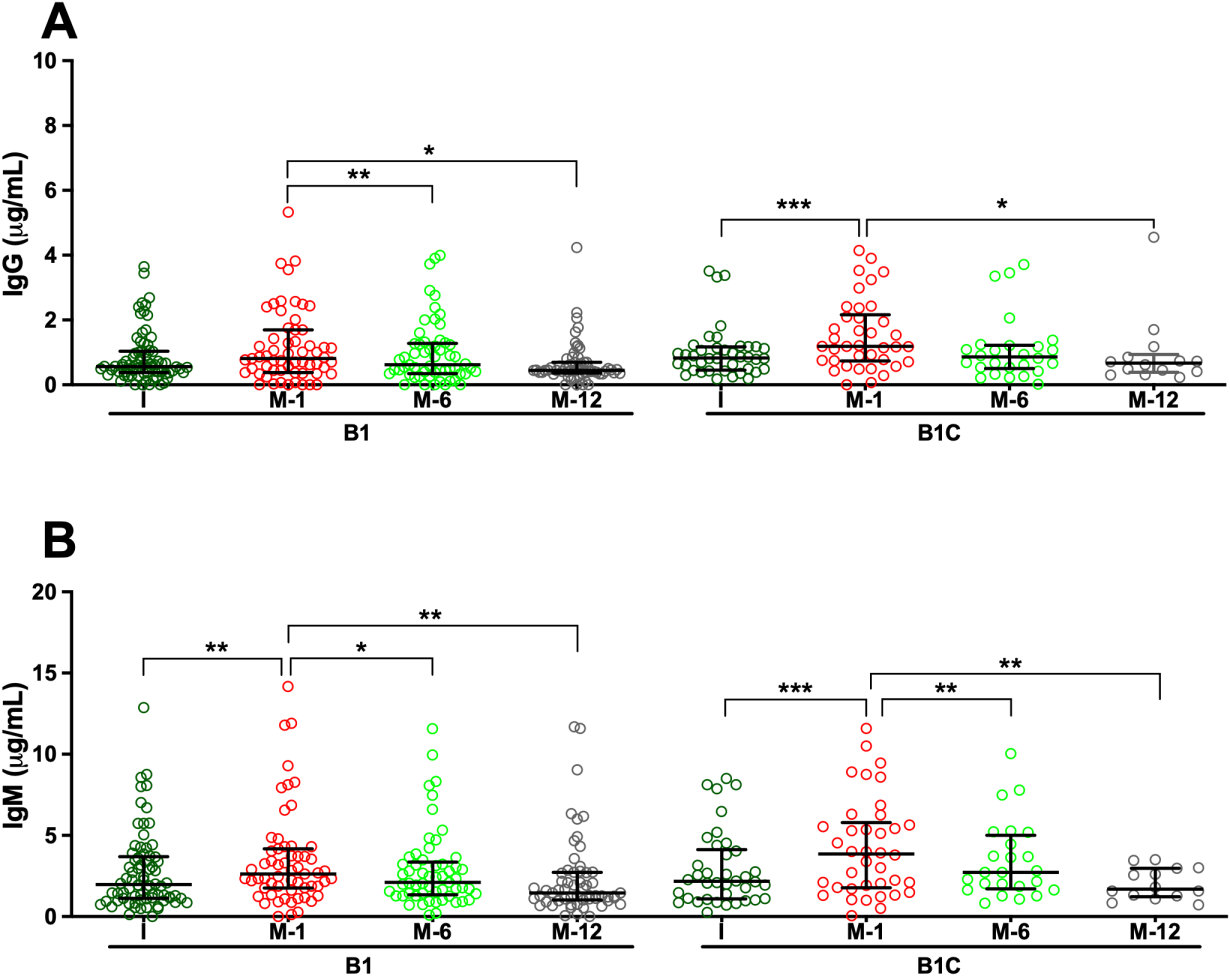
Serum antibodies against the hyaluronan fragment HA84 (42 disaccharide units) measured by ELISA. **(A)** IgG antibodies. **(B)** IgM antibodies. Longitudinal analyses are shown for cohorts B1 (*de novo* BHV implantation) and B1C (*de novo* MHV or CABG), with serum samples collected before surgery (I) and at 1, 6, and 12 months post-operatively (M-1, M-6, and M-12). The results are expressed in μg/mL as median values with interquartile ranges (25th–75th percentiles). (*p < 0.05; **p < 0.01; ***p < 0.001).

Analysis of serum samples by ELISA revealed that all patients, regardless of clinical group, presented measurable IgG and IgM antibodies directed against HA fragments of varying lengths, specifically HA2, HA24, and HA84, at the time of study inclusion. Overall, median IgG antibody titers were comparable across cohorts, with limited statistically significant intergroup differences (**Supplementary Tables S1**-**S3**). For anti-HA2 IgG, median concentrations in the pre-surgery (I) samples of *de novo* BHV recipients (Group B1) were significantly higher than in those observed in long-term BHV recipients without SVD (Group B2; p < 0.05) (**Figure 1A**, **Supplementary Tables S1** and **S7**). Similarly, median anti-HA24 IgG concentration in I samples of Group B1 was significantly higher than that observed in patients with SVD (Group A; p < 0.001) and in long-term BHV recipients without SVD (Group B2; p < 0.001) (**Figure 2A**; **Supplementary Tables S2** and **S8**). Antibodies directed against HA84 were evaluated only in Groups B1 and B1C. In this comparison, median anti-HA84 IgG levels in Group B1 were lower than those in Group B1C, although this difference did not reach statistical significance (*p* = 0.06) (**Figure 3A**; **Supplementary Table S3**). No other significant baseline differences were observed between Group B1 and the corresponding I sample from the control group (Group B1C) across the HA fragments tested. Statistically significant differences in the initial samples of anti-HA IgM antibody responses across cohorts were observed for anti-HA2 and anti-HA24 antibodies (**Supplementary Tables S4** and **S5**). The long-term MHV or CABG control groups (Group B2C) presented significantly lower anti-HA2 IgM titers than Groups B1 (p < 0.001) and B1C (p < 0.001) (**Figure 1B**; **Supplementary Table S9**). In addition, the median anti-HA24 in Group B2C was significantly lower than that observed in long-term BHV recipients without SVD (Group B2; p < 0.01) (**Figure 1B**; **Supplementary Table S10**). No significant differences in baseline anti-HA84 IgM concentrations were detected between Groups B1 and B1C (**Figure 3B**). Likewise, no significant differences in baseline IgM levels against HA2 or HA24 were observed between the pre-surgical samples of these two groups. Across all cohorts, ELISA analysis revealed an inverse relationship between HA fragment length and antibody binding intensity, particularly in IgG measurements. Shorter fragments, such as HA2, presented the strongest antibody signals, whereas the reactivity decreased progressively with increasing oligomer length, reaching minimal levels for HA84 (**Figures 1**-**3**; **Supplementary Tables S1**-**S6**).

### 3.2 Postoperative kinetics of anti-HA antibodies by ELISA

To investigate the kinetics of anti-HA antibody responses following cardiac surgery, serial serum samples from *de novo* BHV recipients (Group B1) and their controls (Group B1C) were analyzed longitudinally by ELISA. In Group B1 patients, *de novo* BHV implantation was followed by a significant postoperative increase in both IgG and IgM antibodies against all three HA fragments tested (HA2, HA24, and HA84) (**Figures 1**-**3**). One month after surgery, the most prominent increase occurred when antibody levels against most oligosaccharides peaked. For the smallest fragment, HA2, IgG concentration increased significantly at one month (*p* < 0.05 M1 vs. I) (**Figure 1A**; **Supplementary Tables S1** and **S11**). IgM antibodies followed a similar trend, peaking at one month (*p* < 0.05 M1 vs. I) and declining thereafter (*p* < 0.05 M12 vs. M1) (**Figure 1**; **Supplementary Tables S4** and **S12**). For the intermediate-length fragment HA24, both IgG and IgM antibodies rose sharply one month after surgery (both *p* < 0.001 M1 vs. I) (**Figure 2**; **Supplementary Tables S2**, **S5**, **S13**, and **S14**), declining thereafter (*p* < 0.001 M6 vs. M1, M12 vs. M1, M12 vs. M6) (**Figure 2**; **Supplementary Tables S2**, **S5**, **S13** and **S14**). Reactivity to the longest fragment, HA84, was consistently lower in magnitude and more transient. IgG antibodies showed a modest increase at one month, which did not reach statistical significance (*p* = 0.08 M1 vs. I), while IgM antibodies augmented significantly at one month (*p* < 0.01 M1 vs. I), with both isotypes returning to baseline thereafter (**Figure 3**; **Supplementary Tables S3**, **S6**, **S15**, and **S16**).

In the surgical control group (Group B1C), which included patients who underwent MHV or CABG, the postoperative dynamics of anti-HA antibodies closely mirrored those observed in BHV recipients. ELISA analysis revealed a significant increase in IgG and IgM antibodies against all HA fragments one month after surgery, followed by a gradual return toward baseline levels over the subsequent months in most cases (**Figures 1**-**3**). For the smallest fragment, HA2, IgG concentration increased significantly at one month post-surgery (*p* < 0.01 M1 vs. I) (**Figure 1**; **Supplementary Tables S1** and **S17**), and a similar pattern was observed for IgM (*p* < 0.001 M1 vs. I) (**Figure 1**; **Supplementary Tables S2** and **S18**). Likewise, IgG anti-HA24 titers increased at one month (*p* < 0.001 M1 vs. I), and peaked at twelve months (*p <* 0.05 M12 vs. I) (**Figure 2**; **Supplementary Tables S2** and **S19**). IgM anti-HA24 antibodies also rose sharply at one month (*p* < 0.001 M1 vs. I) before gradually declining thereafter (*p* < 0.001 M6 vs. M1 and M12 vs. M1) (**Figure 2**; **Supplementary Tables S5** and **S20**). Reactivity to the longest fragment, HA84, was again modest in magnitude and short-lived. IgG and IgM antibodies peaked one month after surgery (both *p* < 0.001 M1 vs. I) and returned to near-pre-surgical levels by six months (IgG *p* < 0.05 M12 vs. M1; IgM *p* < 0.01 M6 vs. M1 and M12 vs. M1) (**Figure 3**; **Supplementary Tables S3**, **S6**, **S21**, and **S22**). Collectively, these results demonstrate that patients who undergo cardiac surgery without xenogeneic tissue implantation exhibit a postoperative surge in anti-HA antibodies comparable to that observed in BHV recipients.

### 3.3 Anti-HA antibody specificity of PGA

**Figure 4:**
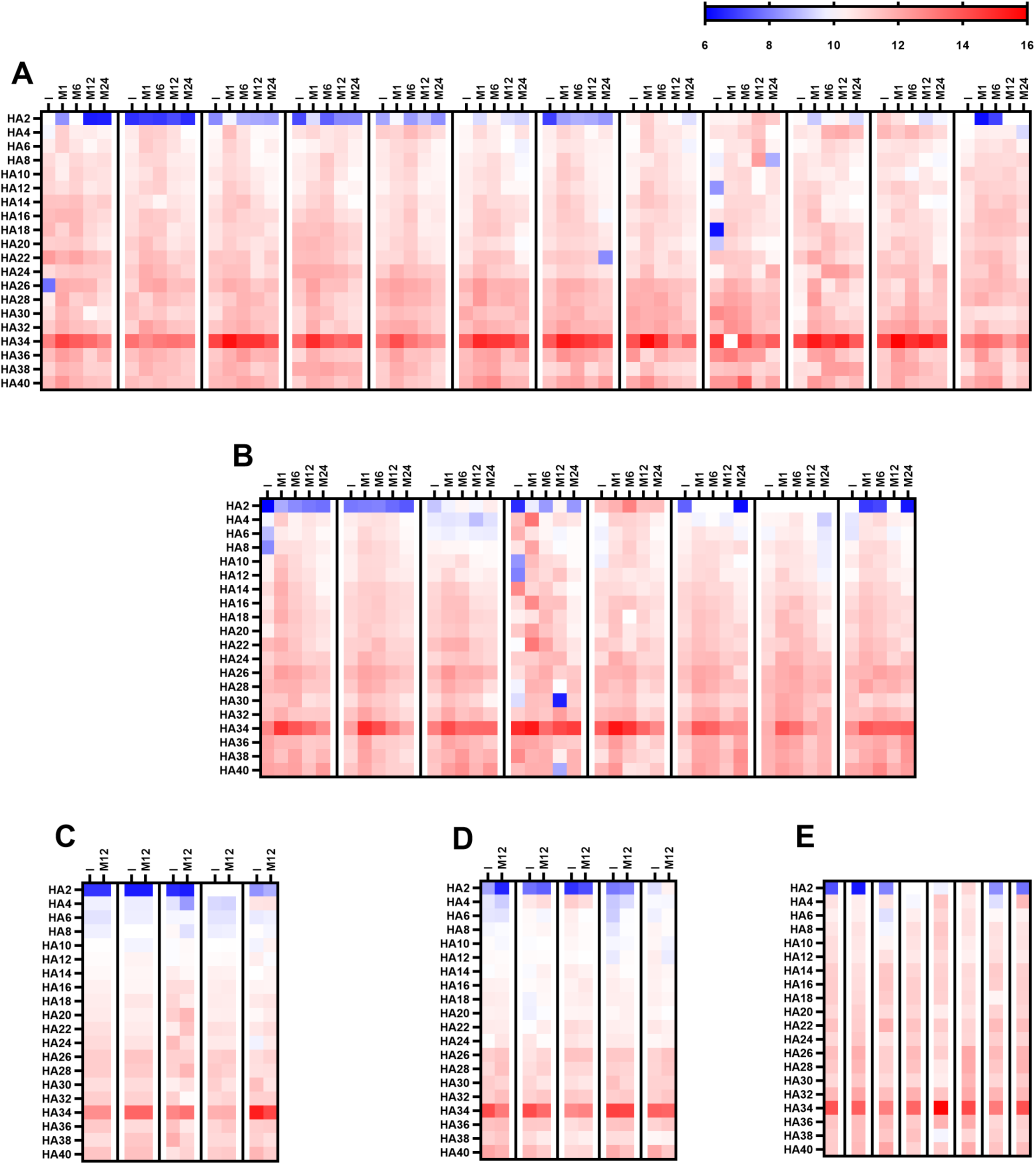
Antibody recognition of HA oligosaccharides assessed via PGA analysis. The heatmap depicts log2 reactivity against 20 structurally defined HA oligosaccharides, ranging from the disaccharide unit (HA2) to 20 repeating disaccharide units (HA40). **(A)** Longitudinal analysis for Group B1 (*de novo* BHV implantation; n = 12) with serum samples collected before surgery (I) and at 1, 6, 12, and 24 months post-surgery (M-1, M-6, M-12, and M-24). **(B)** Longitudinal analysis for Group B1C (*de novo* MHV or CABG; n = 8) with samples collected at the same time points as those for Group B1. **(C)** Baseline and 12-month (M-12) analysis for Group B2 (≥4 years post-BHV without SVD). **(D)** Baseline and 12-month (M-12) analysis for Group B2C (≥4 years post-MHV implantation or CABG). **(E)** serum sample obtained from Group A (SVD) patients. The color scale applies to all the heatmaps.

A representative subset of serum samples from different study cohorts (Group A: 8; Group B1: 12; Group B1C: 8; Group B2: 5; and Group B2C: 5) was analyzed via PGA technology. This high-resolution platform differs substantially from ELISA in antigen immobilization, epitope density, and conformational constraints, and differences in fragment-length–dependent antibody recognition between these assays were anticipated. PGA enabled the simultaneous assessment of antibody binding to 20 structurally defined HA fragments, ranging from the disaccharide unit (HA2) to longer oligomers containing up to 20 repeating disaccharide units (HA40). Overall, detectable antibody binding was observed across most HA fragments tested in the initial sample, confirming the widespread presence of circulating antibodies recognizing HA epitopes of varying oligomer lengths (**Figures 4A–E**). However, a striking difference emerged when these results were compared with the ELISA results. Whereas ELISA revealed potent antibody binding to the shortest fragment, HA2, and PGA showed negligible or undetectable reactivity toward HA2 in nearly all the samples (**Figures 4A–E**). Among the entire panel of HA oligosaccharides tested, HA34 (corresponding to 17 repeating disaccharide units) consistently presented the strongest antibody reactivity in the initial sample across all patient groups (**Figures 4A–E**). This dominant recognition pattern was evident in both BHV recipients and cardiac surgery controls, regardless of cohort or clinical status, and remained across all time points.

In *de novo* BHV recipients (Group B1), PGA profiling revealed a postoperative increase in antibody reactivity across nearly all HA oligomers, with the most pronounced increase occurring one month after valve implantation (**Figure 4A**). This increased reactivity persisted through month six before gradually returning to baseline levels by the end of follow-up (**Figure 4A**). The most substantial and sustained response was directed against HA34 (17 disaccharide units), whose fluorescence intensity remained markedly elevated throughout.

A nearly identical kinetic pattern was observed among the PGAs of cardiac surgery controls (Group B1C; **Figure 4B**). These patients exhibited a parallel postoperative increase in antibody binding to HA fragments, including HA34, peaking at one month and gradually declining thereafter (**Figure 4B**). In contrast, antibody reactivity remained stable in long-term cohorts of BHV recipients (Group B2; **Figure 4C**) and other cardiac surgery controls (Group B2C; **Figure 4D**), with no significant differences between baseline and follow-up samples. The congruence between the novo BHV recipients and surgical controls in the PGA analysis supports the ELISA data, indicating that anti-HA antibody induction reflects a generalized postoperative immune response rather than one specific to bioprosthetic valve materials.

## 4 Discussion

This study expands upon the multicenter Translink investigation, which previously identified antibodies against the xenogeneic glycans αGal and Neu5Gc as contributors to BHV calcification and structural deterioration (5). In this focused subgroup analysis, we investigated humoral immune responses directed against HA, a ubiquitous extracellular matrix glycosaminoglycan with well-established immunomodulatory properties and involvement in native valve calcification and xenogeneic reactivity (10, 11). Using a combination of ELISA and PGA assays, we characterized the presence, kinetics, and fragment-length specificity of anti-HA antibodies in patients undergoing cardiac surgery. Together, these complementary approaches revealed overlapping yet distinct patterns of HA-directed immunity, underscoring the complexity of fragment-dependent immune recognition in this clinical setting.

Like antibodies against xenogeneic glycans such as αGal and Neu5Gc, anti-HA antibodies appear to represent a maintained feature of the natural antibody repertoire (18). At baseline, all patient cohorts—including the SVD (Group A), preoperative (Groups B1 and B1C), and long-term postoperative (Groups B2 and B2C) cohorts—display measurable levels of IgG and IgM antibodies recognizing HA fragments of varying oligomer lengths. This pattern aligns with findings in nonhuman primates and rodents, in which anti-HA activity is detectable under physiological conditions and increases following xenogeneic exposure (11). These observations suggest that HA-specific immune recognition is an evolutionarily conserved element of the natural antibody pool, likely shaped by both microbial and self-derived stimuli. Pathogenic bacteria such as *Streptococcus pyogenes* and *Streptococcus zooepidemicus* express HA capsules identical to those of mammalian HA (19, 20), and can induce cross-reactive antibodies through molecular mimicry (21). Simultaneously, continuous extracellular matrix turnover generates HA fragments that act as endogenous danger signals (14), sustaining low-level immune surveillance of self-glycans and contributing to an evolutionarily conserved balance between tolerance and immune vigilance (22, 23).

At the time of study inclusion, we observed modest but statistically significant differences in anti-HA antibody levels across specific clinical groups. These differences were fragment- and isotype-selective rather than global and did not follow a pattern consistent with sustained xenogeneic immune activation. Notably, baseline anti-HA24 IgG levels were higher in patients sampled prior to *de novo* cardiac surgery (Group B1) compared with patients with established SVD (Group A) and long-term BHV recipients without SVD (Group B2), while no difference was observed between Group B1 and the corresponding pre-surgical control group (B1C). These findings suggest that baseline variability in anti-HA antibodies reflects the preoperative inflammatory and remodeling state rather than prior exposure to bioprosthetic material. Patients awaiting valve replacement or coronary revascularization frequently exhibit chronic inflammation, active extracellular matrix remodeling, and increased tissue stress, all of which may promote endogenous HA fragmentation and immune exposure prior to surgery (24). In contrast, long-term surgical cohorts—particularly non-xenogeneic controls (Group B2C)—displayed lower baseline anti-HA2 IgM levels, consistent with reduced innate-like humoral activation in the absence of ongoing tissue injury. Collectively, these findings reinforce the notion that anti-HA antibodies are constitutive and dynamically modulated by tissue context rather than primarily driven by xenogeneic exposure.

After cardiac surgery, both BHV recipients and cardiac surgical controls (MHV or CABG) exhibited a transient surge in anti-HA antibody titers, peaking at one month and returning toward baseline by six to twelve months. The similar magnitude and kinetics between cohorts indicate that this response is not xenogeneic, reflecting matrix-derived immune activation following surgical trauma, ischemia–reperfusion injury, and inflammation, which drive the enzymatic degradation of high-molecular-weight HA into immunogenic fragments (14, 24, 25). Hence, elevated postoperative anti-HA antibodies likely reflect the systemic inflammatory response and generalized tissue remodeling and repair processes associated with cardiac surgery rather than a xenograft-specific immune response (26, 27). The subsequent normalization of anti-HA antibody levels in long-term cohorts suggests that HA-directed humoral responses do not remain chronically amplified. However, in the absence of healthy controls, it remains unclear whether these stabilized levels fully correspond to physiological ranges in the general population.

Analysis of anti-HA antibodies via both ELISA and a PGA demonstrated that fragment length significantly influences antibody binding. However, the two methods yielded opposite trends. ELISA consistently detected greater antibody binding to short fragments, such as HA2 (one disaccharide unit), whereas PGA identified HA34 (17 disaccharide units) as the dominant immunogenic oligomer across all cohorts. Part of this divergence can be attributed to technical differences in antigen presentation. End-immobilized oligosaccharides via reductive amination in PGA, which disrupts ring structures critical for antibody recognition of very short oligosaccharides such as HA2, versus multivalent HA–PAA conjugates in ELISA (28–30). However, this discrepancy likely also reflects biologically meaningful differences in antibody populations. Anti-HA antibodies are unlikely to constitute a single, uniform population. Rather, they comprise a spectrum of antibody specificities that differ in affinity, epitope recognition, and dependence on HA conformation and polymer length (31). ELISA captures a broad repertoire of low- and moderate-affinity antibodies that recognize small HA epitopes, such as HA2, suggesting abundant innate-like or cross-reactive specificities. In contrast, PGA selectively highlights higher-affinity, conformation-sensitive antibodies that preferentially recognize intermediate-length HA fragments. Similar assay-dependent divergence has been reported (11), supporting the notion that HA2- and HA34-directed antibodies represent distinct yet complementary components of the anti-HA humoral response.

**Figure 5:**
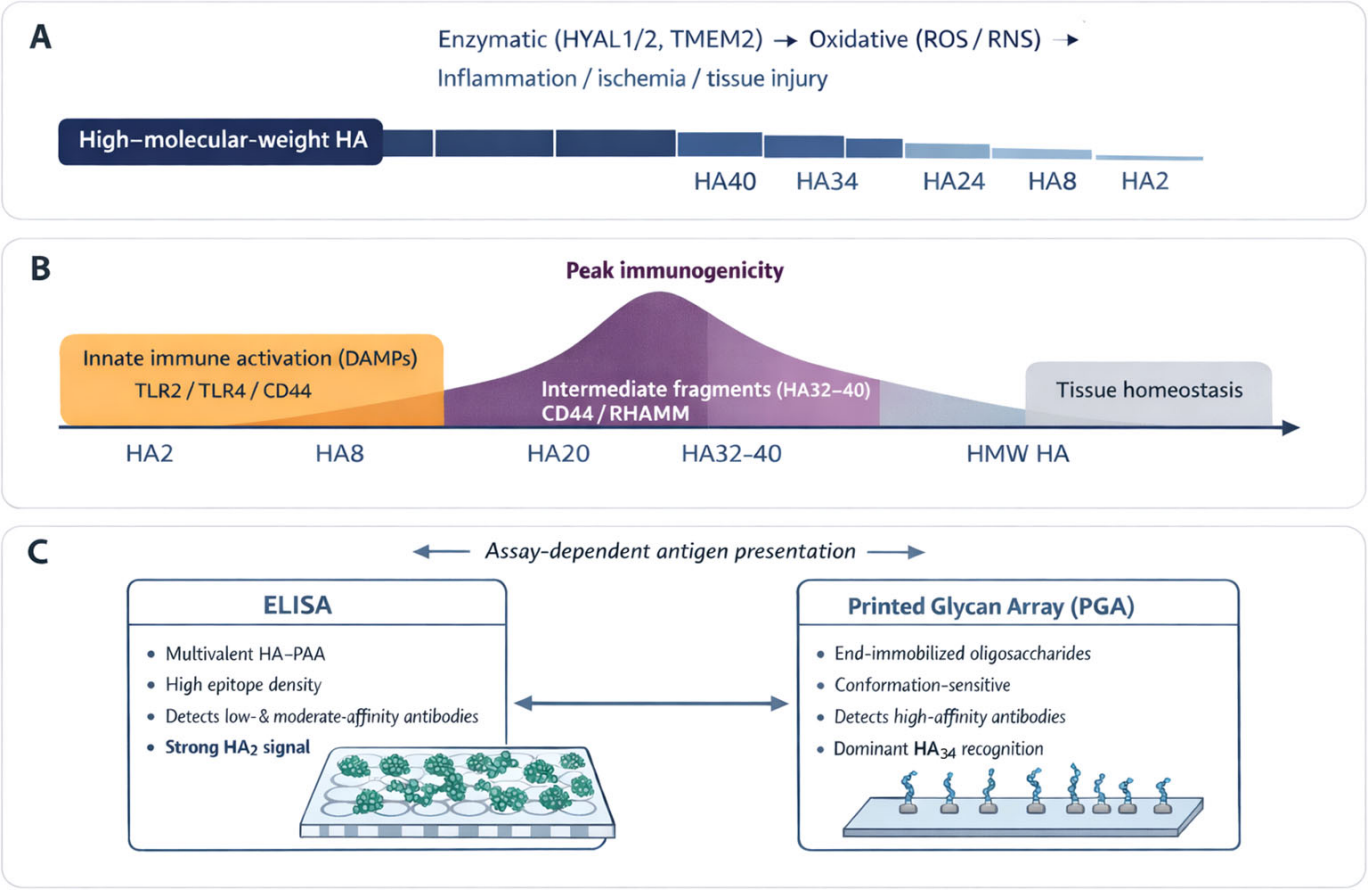
Fragment-length-dependent generation, function, and immune recognition of hyaluronan (HA). **(A)** During tissue injury, inflammation, and remodeling, high–molecular–weight HA is degraded by hyaluronidases and oxidative processes, generating a spectrum of HA fragments rather than a random distribution. **(B)** HA fragment size determines biological activity. Short fragments (HA2–HA20) primarily function as damage-associated molecular patterns, activating innate immune pathways, whereas intermediate fragments (HA32–HA40) more efficiently engage receptors such as CD44 and RHAMM, promoting cell migration, angiogenesis, and matrix remodeling. **(C)** Antibody recognition of HA is fragment-length– and assay-dependent. ELISA preferentially detects abundant, low- to moderate-affinity antibodies that bind short fragments such as HA2, while PGA analysis highlights higher-affinity, conformation-sensitive antibodies that recognize intermediate-length fragments, particularly HA34.

Mechanistically, the reactivity profile observed in ELISA and PGA is consistent with current understanding of HA biology (**Figure 5**). *In vivo*, enzymatic (hyaluronidases) and non-enzymatic (oxidative) HA degradation generate a spectrum of fragment sizes rather than a linear distribution, with certain size ranges, particularly intermediate-length fragments, preferentially produced under inflammatory conditions (24). Shorter HA oligosaccharides (HA8–HA20, 4–10 disaccharide units) are well characterized as DAMPs that activate innate immune signaling through TLR2, TLR4, and CD44 (14, 24). In contrast, intermediate fragments HA32–HA40 (16–20 disaccharide units) exhibit different profiles, binding more stably to receptors such as CD44 and RHAMM and enhancing cell migration, proliferation, angiogenesis, and extracellular matrix remodeling (32–34). In addition to functional differences, short oligomers such as HA2 are potent activators of the innate immune response. However, their limited length reduces their ability to engage multiple receptor sites simultaneously, thereby diminishing their capacity to trigger prolonged or coordinated signaling cascades. In contrast, the intermediate size confers sufficient length for multivalent receptor engagement and B-cell receptor crosslinking (35), remaining short enough to be released during normal extracellular turnover. Thus, HA fragments within this size spectrum appear to occupy a key immunological niche, large enough to elicit structured antigenic recognition but reflective of physiological tissue dynamics that are transiently impaired under stressful conditions such as cardiac surgery. HA34 fragments may therefore represent an evolutionarily conserved signal linking tissue integrity and humoral surveillance, balancing immune tolerance with vigilance toward homeostatic change (36).

This study does not establish a causal relationship between anti-HA antibodies and SVD. However, HA influences native valve calcification through CD44-dependent pathways (10), and stabilized anti-HA responses may act in concert with other immune and remodeling mechanisms over time. It is therefore likely that anti-HA antibodies alone are insufficient predictors of BHV degeneration, underscoring the need for multibiomarker approaches integrating humoral immunity, matrix remodeling, and inflammatory pathways. On the other hand, these findings have broader implications beyond cardiac surgery. HA is widely used in xenogeneic cosmetic, dermatologic, and biomedical products and is often assumed to be immunologically inert. Demonstration of pre-existing anti-HA antibodies and fragment-size–dependent immunogenicity raises the possibility that certain HA preparations, particularly if they are enriched in intermediate-length fragments, could boost undesirable immune responses. Selection or engineering of HA products to avoid size ranges associated with peak antibody recognition may help mitigate this risk.

The strengths of the study include the use of two complementary immunoassays, which together provided both breadth and fine specificity of the anti-HA response, and its embedding within the Translink cohort, which ensured robust clinical characterization. Limitations include the observational design, which precludes causal inference; the absence of a healthy control group, which prevents evaluation of whether the stabilized levels of anti-HA antibodies fully correspond to physiological ranges in the general population; the technical constraints of PGA, which likely underestimate the recognition of very short fragments; and the smaller sample size of the PGA subset of patients. Tissue-level correlations of HA degradation and calcification were unavailable, limiting mechanistic interpretation.

In conclusion, this study demonstrates that anti-HA antibodies are constitutively present in humans and are dynamically regulated following cardiac surgery, with a transient postoperative amplification that is independent of prosthesis type. ELISA analyses performed in a substantially larger cohort revealed robust IgG and IgM responses to short HA fragments, such as HA2, highlighting widespread innate-like HA recognition. PGA profiling identified intermediate-length fragments, particularly HA34, as conserved, immunodominant targets of higher-affinity antibody responses. Together, these findings extend the Translink paradigm beyond xenogeneic glycan immunity to include extracellular matrix–derived glycan immunity and support a fragment-length–dependent model of HA immunogenicity. Anti-HA antibodies, including both HA2- and HA34-directed specificities, may serve as biomarkers of tissue inflammation and remodeling and represent one component of a broader immune landscape relevant to bioprosthetic valve durability and other HA-based biomedical applications.

## Data Availability

The raw data supporting the conclusions of this article will be made available by the authors, without undue reservation.
