# Supplementary material for: Humoral immune responses to hyaluronan oligosaccharides in patients undergoing prosthetic valve surgery
Source: Front Immunol. 2026 Feb 18;17:1762676. doi: 10.3389/fimmu.2026.1762676 (PMC12957271; doi:10.3389/fimmu.2026.1762676)
Supplement: Supplementary file 1 [file Table1.docx]

Supplementary Material

# Supplementary Tables

**Table S1. Anti-HA2 IgG antibodies by Group and Time points**

| *Group* | *Time point* | *n* | *Median (ng/µL)* | *Range (min–max)* |
| --- | --- | --- | --- | --- |
| A | Initial sample | 29 | 2.14 | 0.18–11.63 |
| B1 | Initial sample (pre-surgery) | 133 | 2.97 | 0–26.77 |
| B1 | 1 month | 116 | 3.97 | 0–26.34 |
| B1 | 6 months | 103 | 3.73 | 0–24.78 |
| B1 | 12 months | 92 | 4.84 | 0.01–25.26 |
| B1 | 24 months | 13 | 2.65 | 0.45-19.62 |
| B1C | Initial sample (pre-surgery) | 43 | 1.94 | 0.39–26.85 |
| B1C | 1 month | 41 | 2.92 | 0.37–31.13 |
| B1C | 6 months | 27 | 2.36 | 0-21.65 |
| B1C | 12 months | 14 | 2.72 | 0-10.76 |
| B2 | Initial sample | 96 | 1.87 | 0.05–20.91 |
| B2C | Initial sample | 35 | 2.00 | 0.05–20.91 |

Table S2. Anti-HA24 IgG antibodies by Group and Time points

| *Group* | *Time point* | *n* | *Median (ng/µL)* | *Range (min–max)* |
| --- | --- | --- | --- | --- |
| A | Initial sample | 29 | 0.47 | 0.06–2.87 |
| B1 | Initial sample (pre-surgery) | 133 | 1.14 | 0.14–23.87 |
| B1 | 1 month | 114 | 2.34 | 0–28.99 |
| B1 | 6 months | 101 | 1.75 | 0–25.81 |
| B1 | 12 months | 92 | 1.06 | 0.21–23.64 |
| B1 | 24 months | 13 | 1.34 | 0.27-4.23 |
| B1C | Initial sample (pre-surgery) | 43 | 0.86 | 0.03–20.75 |
| B1C | 1 month | 41 | 1.38 | 0–26.31 |
| B1C | 6 months | 27 | 1.19 | 0.26-14.85 |
| B1C | 12 months | 14 | 1.76 | 0.46-23.75 |
| B2 | Initial sample | 96 | 0.69 | 0–10.63 |
| B2C | Initial sample | 35 | 0.78 | 0–4.55 |

**Table S3.** **Anti-HA84 IgG antibodies by Group and Time points**

| *Group* | *Time point* | *n* | *Median (ng/µL)* | *Range (min–max)* |
| --- | --- | --- | --- | --- |
| B1 | Initial sample (pre-surgery) | 74 | 0.56 | 0–3.64 |
| B1 | 1 month | 59 | 0.81 | 0–5.33 |
| B1 | 6 months | 57 | 0.62 | 0–3.99 |
| B1 | 12 months | 58 | 0.45 | 0–4.24 |
| B1C | Initial sample (pre-surgery) | 40 | 0.83 | 0.18–3.51 |
| B1C | 1 month | 38 | 1.19 | 0–4.14 |
| B1C | 6 months | 26 | 0.86 | 0.02-3.71 |
| B1C | 12 months | 14 | 0.67 | 0.23-4.56 |
|  |  |  |  |  |
|  |  |  |  |  |
|  |  |  |  |  |
|  |  |  |  |  |

Table S4. Anti-HA2 IgM antibodies by Group and Time points

| *Group* | *Time point* | *n* | *Median (ng/µL)* | *Range (min–max)* |
| --- | --- | --- | --- | --- |
| A | Initial sample | 29 | 6.67 | 0.99–19.97 |
| B1 | Initial sample (pre-surgery) | 131 | 8.09 | 0.55–43.47 |
| B1 | 1 month | 114 | 9.49 | 1.00–40.89 |
| B1 | 6 months | 102 | 8.22 | 0–43.41 |
| B1 | 12 months | 92 | 8.63 | 0.66–44.68 |
| B1 | 24 months | 13 | 7.81 | 2.46-27.58 |
| B1C | Initial sample (pre-surgery) | 43 | 8.80 | 0.38–24.30 |
| B1C | 1 month | 41 | 9.81 | 1.57–28.04 |
| B1C | 6 months | 29 | 8.32 | 0-28.87 |
| B1C | 12 months | 14 | 8.37 | 0.42-23.49 |
| B2 | Initial sample | 96 | 7.09 | 0.45–40.48 |
| B2C | Initial sample | 35 | 4.54 | 0.94–14.72 |

**Table S5. Anti-HA24 IgM antibodies by Group and Time points**

| *Group* | *Time point* | *n* | *Median (ng/µL)* | *Range (min–max)* |
| --- | --- | --- | --- | --- |
| A | Initial sample | 29 | 2.28 | 0.34–17.68 |
| B1 | Initial sample (pre-surgery) | 131 | 2.50 | 0.27–36.91 |
| B1 | 1 month | 114 | 4.15 | 0–32.39 |
| B1 | 6 months | 102 | 2.84 | 0–35.32 |
| B1 | 12 months | 92 | 2.12 | 0.38–35.31 |
| B1 | 24 months | 13 | 1.92 | 0.59-5.77 |
| B1C | Initial sample (pre-surgery) | 43 | 2.68 | 0.63–35.14 |
| B1C | 1 month | 41 | 3.92 | 1.11–28.72 |
| B1C | 6 months | 27 | 2.92 | 0.99-8.94 |
| B1C | 12 months | 14 | 2.49 | 0.75-15.53 |
| B2 | Initial sample | 96 | 2.92 | 0.25–21.50 |
| B2C | Initial sample | 35 | 1.46 | 0.28–8.45 |

Table S6. Anti-HA84 IgM antibodies by Group and Time points

| *Group* | *Time point* | *n* | *Median (ng/µL)* | *Range (min–max)* |
| --- | --- | --- | --- | --- |
| B1 | Initial sample (pre-surgery) | 74 | 1.97 | 0–12.88 |
| B1 | 1 month | 59 | 2.63 | 0–14.18 |
| B1 | 6 months | 57 | 2.11 | 0.09–11.57 |
| B1 | 12 months | 58 | 1.45 | 0–11.69 |
| B1C | Initial sample (pre-surgery) | 38 | 2.17 | 0.26–8.51 |
| B1C | 1 month | 38 | 3.86 | 0.06–11.60 |
| B1C | 6 months | 24 | 2.72 | 0.82-10.04 |
| B1C | 12 months | 14 | 1.70 | 0.72-3.51 |

**Table S7. Dunn’s multiple comparisons test for anti-HA2 IgG antibodies**

| *Dunn's* | *Mean rank difference* | *Adjusted P Value* |
| --- | --- | --- |
| B1-I vs. B1C-I | 31.26 | 0.6663 |
| B1-I vs. B2 | 42.41 | 0.0111 |
| B1-I vs. B2C | 39.67 | 0.3160 |
| B1-I vs. A | 30.65 | >0.9999 |
| B1C-I vs. B2 | 11.16 | >0.9999 |
| B1C-I vs. B2C | 8.411 | >0.9999 |
| B1C-I vs. A | -0.6018 | >0.9999 |
| B2 vs. B2C | -2.744 | >0.9999 |
| B2 vs. A | -11.76 | >0.9999 |
| B2C vs. A | -9.013 | >0.9999 |

**Table S8. Dunn’s multiple comparisons test for anti-HA24 IgG antibodies**

| *Dunn's* | *Mean rank difference* | *Adjusted P Value* |
| --- | --- | --- |
| B1-I vs. B1C-I | 37.84 | 0.2637 |
| B1-I vs. B2 | 55.07 | 0.0002 |
| B1-I vs. B2C | 46.90 | 0.1104 |
| B1-I vs. A | 88.35 | <0.0001 |
| B1C-I vs. B2 | 17.22 | >0.9999 |
| B1C-I vs. B2C | 9.058 | >0.9999 |
| B1C-I vs. A | 50.50 | 0.3049 |
| B2 vs. B2C | -8.165 | >0.9999 |
| B2 vs. A | 33.28 | >0.9999 |
| B2C vs. A | 41.44 | 0.8930 |

**Table S9. Dunn’s multiple comparisons test for anti-HA2 IgM antibodies**

| *Dunn's* | *Mean rank difference* | *Adjusted P Value* |
| --- | --- | --- |
| B1-I vs. B1C-I | -18.30 | >0.9999 |
| B1-I vs. B2 | 20.21 | >0.9999 |
| B1-I vs. B2C | 72.15 | 0.0009 |
| B1-I vs. A | 32.83 | 0.9760 |
| B1C-I vs. B2 | 38.51 | 0.2977 |
| B1C-I vs. B2C | 90.45 | 0.0004 |
| B1C-I vs. A | 51.13 | 0.2756 |
| B2 vs. B2C | 51.94 | 0.0644 |
| B2 vs. A | 12.62 | >0.9999 |
| B2C vs. A | -39.32 | >0.9999 |

**Table S10. Dunn’s multiple comparisons test for anti-HA24 IgM antibodies**

| *Dunn's* | *Mean rank difference* | *Adjusted P Value* |
| --- | --- | --- |
| B1-I vs. B1C-I | -2.157 | >0.9999 |
| B1-I vs. B2 | -10.74 | >0.9999 |
| B1-I vs. B2C | 46.78 | 0.1090 |
| B1-I vs. A | 29.91 | >0.9999 |
| B1C-I vs. B2 | -8.584 | >0.9999 |
| B1C-I vs. B2C | 48.93 | 0.2601 |
| B1C-I vs. A | 32.07 | >0.9999 |
| B2 vs. B2C | 57.52 | 0.0256 |
| B2 vs. A | 40.65 | 0.4695 |
| B2C vs. A | -16.87 | >0.9999 |

**Table S11. Tukey’s multiple comparisons test for anti-HA2 IgG antibodies in Group B1**

| *Tukey's* | *Mean difference* | *Adjusted P Value* |
| --- | --- | --- |
| I vs. M-1 | -0.04340 | 0.0178 |
| I vs. M-6 | -0.03135 | 0.0833 |
| I vs. M-12 | -0.02586 | 0.1826 |
| I vs. M-24 | -0.01025 | 0.9620 |
| M-1 vs. M-6 | 0.01418 | 0.3027 |
| M-1 vs. M-12 | 0.02547 | 0.5358 |
| M-1 vs. M-24 | 0.03752 | 0.6021 |
| M-6 vs. M-12 | 0.01998 | 0.0546 |
| M-6 vs. M-24 | 0.02192 | 0.6941 |
| M-12 vs. M-24 | -0.004465 | 0.9975 |

**Table S12. Tukey’s multiple comparisons test for anti-HA2 IgM antibodies in Group B1**

| *Tukey's* | *Mean difference* | *Adjusted P Value* |
| --- | --- | --- |
| I vs. M-1 | -1.213 | 0.0148 |
| I vs. M-6 | -0.4427 | 0.4982 |
| I vs. M-12 | -0.2432 | 0.7465 |
| I vs. M-24 | -0.9717 | 0.4332 |
| M-1 vs. M-6 | 0.8981 | 0.0888 |
| M-1 vs. M-12 | 1.376 | 0.0266 |
| M-1 vs. M-24 | 1.107 | 0.8971 |
| M-6 vs. M-12 | 0.4203 | 0.4857 |
| M-6 vs. M-24 | 0.08854 | >0.9999 |
| M-12 vs. M-24 | -0.1993 | 0.9981 |

**Table S13. Tukey’s multiple comparisons test for anti-HA24 IgG antibodies in Group B1**

| *Tukey's* | *Mean difference* | *Adjusted P Value* |
| --- | --- | --- |
| I vs. M-1 | -0.1378 | <0.0001 |
| I vs. M-6 | -0.05949 | 0.0049 |
| I vs. M-12 | -0.02169 | 0.6100 |
| I vs. M-24 | 0.03302 | 0.7016 |
| M-1 vs. M-6 | 0.08311 | <0.0001 |
| M-1 vs. M-12 | 0.1535 | <0.0001 |
| M-1 vs. M-24 | 0.1497 | 0.0125 |
| M-6 vs. M-12 | 0.05185 | <0.0001 |
| M-6 vs. M-24 | 0.1233 | 0.0047 |
| M-12 vs. M-24 | 0.08265 | 0.0394 |

**Table S14. Tukey’s multiple comparisons test for anti-HA24 IgM antibodies in Group B1**

| *Tukey's* | *Mean difference* | *Adjusted P Value* |
| --- | --- | --- |
| I vs. M-1 | -0.1285 | <0.0001 |
| I vs. M-6 | -0.004297 | 0.9988 |
| I vs. M-12 | 0.01810 | 0.7717 |
| I vs. M-24 | 0.003878 | 0.9997 |
| M-1 vs. M-6 | 0.1292 | <0.0001 |
| M-1 vs. M-12 | 0.1865 | <0.0001 |
| M-1 vs. M-24 | 0.2895 | 0.0016 |
| M-6 vs. M-12 | 0.05244 | <0.0001 |
| M-6 vs. M-24 | 0.1012 | 0.0018 |
| M-12 vs. M-24 | 0.01630 | 0.7873 |

**Table S15. Tukey’s multiple comparisons test for anti-HA84 IgG antibodies in Group B1**

| *Tukey's* | *Mean difference* | *Adjusted P Value* |
| --- | --- | --- |
| I vs. M-1 | -0.03554 | 0.0809 |
| I vs. M-6 | -0.002651 | 0.9971 |
| I vs. M-12 | 0.003827 | 0.9838 |
| M-1 vs. M-6 | 0.03176 | 0.0034 |
| M-1 vs. M-12 | 0.03712 | 0.0194 |
| M-6 vs. M-12 | 0.008202 | 0.8086 |

**Table S16. Tukey’s multiple comparisons test for anti-HA84 IgM antibodies in Group B1**

| *Tukey's* | *Mean difference* | *Adjusted P Value* |
| --- | --- | --- |
| I vs. M-1 | -0.06609 | 0.0044 |
| I vs. M-6 | -0.005714 | 0.9716 |
| I vs. M-12 | 0.02178 | 0.4876 |
| M-1 vs. M-6 | 0.05386 | 0.0250 |
| M-1 vs. M-12 | 0.08133 | 0.0057 |
| M-6 vs. M-12 | 0.04029 | 0.0376 |

**Table S17. Tukey’s multiple comparisons test for anti-HA2 IgG antibodies in Group B1C**

| *Tukey's* | *Mean difference* | *Adjusted P Value* |
| --- | --- | --- |
| I vs. M-1 | -0.1032 | 0.0018 |
| I vs. M-6 | -0.05904 | 0.1343 |
| I vs. M-12 | -0.05832 | 0.6853 |
| M-1 vs. M-6 | 0.04544 | 0.2370 |
| M-1 vs. M-12 | -0.01319 | 0.9963 |
| M-6 vs. M-12 | -0.03382 | 0.9015 |

**Table S18. Tukey’s multiple comparisons test for anti-HA2 IgM antibodies in Group B1C**

| *Tukey's* | *Mean difference* | *Adjusted P Value* |
| --- | --- | --- |
| I vs. M-1 | -0.07826 | <0.0001 |
| I vs. M-6 | 0.05483 | 0.7676 |
| I vs. M-12 | -0.02468 | 0.7897 |
| M-1 vs. M-6 | 0.1345 | 0.0816 |
| M-1 vs. M-12 | 0.07225 | 0.3091 |
| M-6 vs. M-12 | -0.0009127 | >0.9999 |

**Table S19. Tukey’s multiple comparisons test for anti-HA24 IgG antibodies in Group B1C**

| *Tukey's* | *Mean difference* | *Adjusted P Value* |
| --- | --- | --- |
| I vs. M-1 | -0.1226 | 0.0012 |
| I vs. M-6 | -0.08630 | 0.2834 |
| I vs. M-12 | -0.1015 | 0.3057 |
| M-1 vs. M-6 | 0.09640 | 0.2070 |
| M-1 vs. M-12 | 0.1923 | 0.0143 |
| M-6 vs. M-12 | 0.04714 | 0.5009 |

**Table S20. Tukey’s multiple comparisons test for anti-HA24 IgM antibodies in Group B1C**

| *Tukey's* | *Mean difference* | *Adjusted P Value* |
| --- | --- | --- |
| I vs. M-1 | -0.1583 | 0.0001 |
| I vs. M-6 | 0.01929 | 0.9570 |
| I vs. M-12 | -0.01234 | 0.9525 |
| M-1 vs. M-6 | 0.2031 | <0.0001 |
| M-1 vs. M-12 | 0.1624 | 0.0003 |
| M-6 vs. M-12 | 0.01507 | 0.9452 |

**Table S21. Tukey’s multiple comparisons test for anti-HA84 IgG antibodies in Group B1C**

| *Tukey's* | *Mean difference* | *Adjusted P Value* |
| --- | --- | --- |
| I vs. M-1 | -0.09145 | 0.0016 |
| I vs. M-6 | -0.03374 | 0.2357 |
| I vs. M-12 | -0.01164 | 0.8908 |
| M-1 vs. M-6 | 0.03517 | 0.4692 |
| M-1 vs. M-12 | 0.08110 | 0.0470 |
| M-6 vs. M-12 | 0.01272 | 0.7705 |

**Table S22. Tukey’s multiple comparisons test for anti-HA84 IgM antibodies in Group B1C**

| *Tukey's* | *Mean difference* | *Adjusted P Value* |
| --- | --- | --- |
| I vs. M-1 | -0.1324 | <0.0001 |
| I vs. M-6 | -0.05884 | 0.0596 |
| I vs. M-12 | -0.03785 | 0.6206 |
| M-1 vs. M-6 | 0.07147 | 0.0052 |
| M-1 vs. M-12 | 0.1322 | 0.0017 |
| M-6 vs. M-12 | 0.06229 | 0.1635 |
